# Determinants of Cluster Size in Large, Population-Based Molecular Epidemiology Study of Tuberculosis, Northern Malawi

**DOI:** 10.3201/eid1407.060468

**Published:** 2008-07

**Authors:** Judith R. Glynn, Amelia C. Crampin, Hamidou Traore, Steve Chaguluka, Donex T. Mwafulirwa, Saad Alghamdi, Bagrey M.M. Ngwira, Malcolm D. Yates, Francis D. Drobniewski, Paul E.M. Fine

**Affiliations:** * London School of Hygiene and Tropical Medicine, London, UK; † Karonga Prevention Study, Chilumba, Malawi; ‡ Health Protection Agency, London

**Keywords:** Tuberculosis, HIV, RFLP, tuberculosis transmission, Africa, research

## Abstract

Tuberculosis patients with identical strains of *Mycobacterium tuberculosis* are described as clustered. Cluster size may depend on patient or strain characteristics. In a 7-year population-based study of tuberculosis in Karonga District, Malawi, clusters were defined by using IS*6110* restriction fragment length polymorphism, excluding patterns with <5 bands. Spoligotyping was used to compare strains with an international database. Among 682 clustered patients, cluster size ranged from 2 to 37. Male patients, young adults, and town residents were over-represented in large clusters. Cluster size was not associated with HIV status or death from tuberculosis. Spoligotypes from 9 (90%) of 10 large cluster strains were identical or very similar (1 spacer different) to common spoligotypes found elsewhere, compared with 37 (66%) of 56 of those from nonclustered patients (p = 0.3). Large clusters were associated with factors likely to be related to social mixing, but spoligotypes of common strains in this setting were also common types elsewhere, consistent with strain differences in transmissibility.

## Abstract 2

Both epidemiologic and strain-related factors may contribute to large clusters of tuberculosis patients.

Molecular techniques, in particular restriction fragment length polymorphism (RFLP) based on the IS*6110* insertion element, are used to define clusters of isolates of *Mycobacterium tuberculosis* with identical DNA fingerprints. Many studies have investigated risk factors for clustering, but relatively little is known about the determinants of cluster size (*1*,*2*). The size of clusters could depend on factors favoring transmission or on differences in the strains themselves. *M. tuberculosis* strains found in persons with smear-positive disease, many contacts, or delays in diagnosis and effective treatment are particularly likely to be transmitted. Some strains may be inherently more transmissible than others, perhaps because they are particularly likely to give rise to sputum smear–positive disease, they are associated with a more insidious onset of clinical symptoms (so patients are infectious for longer), or they are more virulent and are therefore more likely to give rise to secondary cases within the period studied (*3*). Large clusters may also be observed if the strain has a particularly stable RFLP pattern; this may be more likely for strains with few bands.

Epidemiologic differences can be explored by examining risk factors for cluster size. Giordano et al. (*1*) hypothesized that cluster size would be related to duration of symptoms. Those researchers found no evidence of this but did find inverse associations with age and HIV status in a population-based study in Texas in the United States. Strain-related differences are likely if the same strains give rise to large clusters in unrelated populations. The ubiquity of the Beijing family of strains has led to speculation that they may be particularly virulent or transmissible (*4*).

In a population-based study of the molecular epidemiology of tuberculosis in northern Malawi, we found that clustering was associated with young age, female sex, area of residence, and, in older adults, HIV positivity (*5*). We explored the determinants of cluster size and the characteristics of the larger clusters.

## Methods

As part of the Karonga Prevention Study, northern Malawi, all persons with suspected tuberculosis at peripheral clinics and the district hospital are seen by project staff. Sputum is collected for smear and culture; lymph node and pleural and peritoneal aspirates are also cultured, when available. Cultures are set up in the project laboratory in Malawi, and those macroscopically consistent with *M. tuberculosis* are sent to the Health Protection Agency Mycobacterium Reference Unit, London, United Kingdom, for species identification and drug resistance testing. HIV testing is conducted after counseling, if consent is given. Patients are treated for tuberculosis according to Malawi government guidelines (*6*).

DNA fingerprinting using IS*6110* RFLP has been conducted on isolates from patients who have been diagnosed since late 1995, following standard procedures (*7*). Patients whose disease was diagnosed up to early 2003 were included in this analysis. RFLP patterns were compared by using computer-assisted (Gelcompar 4.1; Applied Maths, Kortrijk, Belgium) visual comparison. Laboratory error was thought likely if isolates with identical RFLP patterns were isolated on the same day from patients with no known epidemiologic relationship if, in addition, there was no other laboratory evidence of tuberculosis, or if they were the only 2 examples of this RFLP pattern, or if the patients had other isolates with different patterns (*8*). After likely laboratory errors were excluded, RFLP patterns shared by >1 patient were classified as clustered. Some patients had >1 isolate. To define whether a strain was clustered and to determine the size of the cluster, patients were included more than once if they had >1 RFLP pattern. Thereafter, patients were only included once, for their first episode of tuberculosis for which an RFLP result was available.

Spoligotyping (*9*) was performed on at least 2 isolates of clusters containing at least 15 patients, to enable comparison of strains with international databases (*10*,*11*). Changes in the proportion of tuberculosis cases caused by each of these large cluster strains over time was examined, by using the Fisher exact test to compare proportions and the χ^2^ test for linear trend. Spoligotyping was also performed on unique (not clustered) strains from patients with smear-positive tuberculosis in 1998 or 1999, as examples of strains that had apparently not spread in the population; and from all positive cultures from 2002. Previously identified spoligotypes were defined as widespread if the international database described them as both “ubiquitous” and “recurrent,” “common,” or “epidemic.”

Analysis of cluster size excluded unique strains and strains with <5 bands on the RFLP (because patterns with few bands are insufficiently discriminatory). Cluster size was divided into 4 groups (Table 1), and associations with cluster size were determined by using maximum-likelihood ordered logistic regression with the ologit command in STATA (*12*). With this method, the odds ratios calculated represent the summary relative odds of larger clusters compared to smaller clusters across the 4 groups. This method was used in preference to linear regression because cluster size is not normally distributed, and in preference to logistic regression because it avoids arbitrary dichotomization of cluster size. All available risk factors for cluster size were assessed individually (Table 1), and factors that were significant at the 5% level, after adjusting for other factors, or that confounded other variables were retained in the final model. The molecular epidemiologic work of the Karonga Prevention Study was approved by the Malawi National Health Sciences Research Committee and the ethics committee of the London School of Hygiene and Tropical Medicine.

**Table 1:** Associations between patient characteristics and cluster size, *Mycobacterium tuberculosis*, bivariate analysis, Malawi

Characteristic	N	Cluster size, %				Odds ratio (95% CI)*	p value
		2–4 (n = 186)	5–10 (n = 196)	11–20 (n = 173)	>20 (n = 127)		
Age, y							0.05
<25	111	27.9	25.2	25.2	21.6	1	
25–34	266	24.4	26.3	27.8	21.4	1.1 (0.74–1.7)	
35–44	160	26.3	32.5	25.0	16.3	0.87 (0.56–1.3)	
>45	145	33.1	31.7	21.4	13.8	0.66 (0.42–1.0)	
Sex							0.1
F	386	27.2	30.3	26.9	15.0	1	
M	296	26.7	26.7	23.3	23.3	1.2 (0.95–1.6)	
HIV status							1.0
Negative	141	26.2	30.5	23.4	19.9	1	
Positive	288	26.4	28.1	27.8	17.7	1.0 (0.7–1.4)	
Area							0.005
South, near Chilumba	70	38.6	22.9	24.3	14.3	0.51 (0.31–0.84)	
Middle, near Nyungwe	89	20.2	22.5	30.3	27.0	1.2 (0.79–1.9)	
Around Karonga	119	25.2	34.5	21.9	18.5	0.73 (0.48–1.1)	
Karonga central (urban)	209	21.5	25.4	32.5	20.6	1	
Kaporo area	84	35.7	34.5	15.5	14.3	0.46 (0.29–1.1)	
Far north	57	28.1	40.4	21.1	10.5	0.54 (0.32–0.9)	
Outside district	16	26.9	28.9	25.6	18.6	0.41 (0.61–1.1)	
Tuberculosis type							0.6
Smear positive	485	26.2	29.5	25.4	19.0	1	
Smear negative	145	29.7	24.8	27.6	17.9	0.94 (0.68–1.3)	
Extrapulmonary	52	30.8	32.7	19.2	17.3	0.78 (0.47–1.3)	
Previous tuberculosis							0.6
No	628	27.4	28.8	25.8	18.0	1	
Yes	47	27.7	25.5	21.3	25.5	1.2 (0.68–2.0)	
Isoniazid resistance							0.2
No	641	28.1	27.9	25.9	18.1	1	
Yes	39	12.8	43.6	15.4	28.2	1.5 (0.84–2.6)	
Died†							1.0
No	382	26.7	28.3	25.1	19.9	1	
Yes	155	25.8	29.0	26.5	18.7	1.0 (0.72–1.4)	

*Odds ratio from ordered logistic regression. This represents the summary relative odds of larger clusters compared with smaller clusters across the 4 groups. Odds ratios >1 imply that the odds of being in a larger cluster are greater than in the baseline group. CI, confidence interval. †Outcome recorded for 632 patients; those who were lost or transferred while receiving treatment are excluded from this analysis.

## Results

Over the study period, 1,248 cases of culture-positive tuberculosis were diagnosed in patients in Karonga District. RFLP results were available on 1,194 isolates from 1,044 patients. After we excluded 25 isolates because laboratory error was suspected (*8*), there were results for 1,029 patients. Eighty-one had <5 bands so they were excluded. Of the remaining 948 patients, 682 (72%) were clustered and form the basis of this analysis.

Cluster size varied from 2 to 37. The determinants of cluster size are shown in Table 1. Older patients were less likely than younger patients to be in large clusters. Male patients were more likely than female patients to be in large clusters, and there was variation by geographic area. Cluster size was not statistically associated with HIV status, type of tuberculosis, previous tuberculosis, or drug resistance. Patients in small clusters were as likely to die during treatment as those in large clusters. In the multivariate analysis, the results were similar (Table 2), with significant associations with age, sex, and area of residence. The results were unchanged by adjusting for year or for RFLP band number. None of the other factors shown in Table 1 was associated with clustering after we adjusted for possible confounders. Repeating the analysis with different categorizations of cluster size gave similar results (not shown).

**Table 2:** Multivariate analysis of risk factors for larger cluster size, Malawi

Risk factor	Cluster size*		p value
	Odds ratio	95% CI	
Age, y			
<25	1		0.01†
25–34	1.0	0.66–1.5	
35–44	0.76	0.48–1.2	
>45	0.62	0.39–1.0	
Male	1.4	1.0–1.8	0.03
Area			<0.001
South, near Chilumba	0.54	0.33–0.90	
Middle, near Nyungwe	1.3	0.81–2.0	
Around Karonga	0.74	0.50–1.1	
Karonga central (urban)	1		
Kaporo area	0.48	0.30–0.76	
Far north	0.55	0.32–0.92	
Outside district	0.41	0.15–1.1	

*Odds ratios are adjusted for the other factors shown in the table (i.e., the multivariate equation contained age group, sex, and area). CI, confidence interval. †Trend.

All of the large cluster strains (>15 people) were found in at least 4 of the 6 geographic areas of the district, and most were found throughout the district. The distributions of the 4 largest clusters are shown in the Figure. Patients with strains from most of the large clusters were present in the district throughout the study period. Trends over time for strains involving at least 15 people are shown in Table 3. Only 1 strain, kps121, showed statistically significant changes over time; it appeared to be decreasing.

**Figure:**
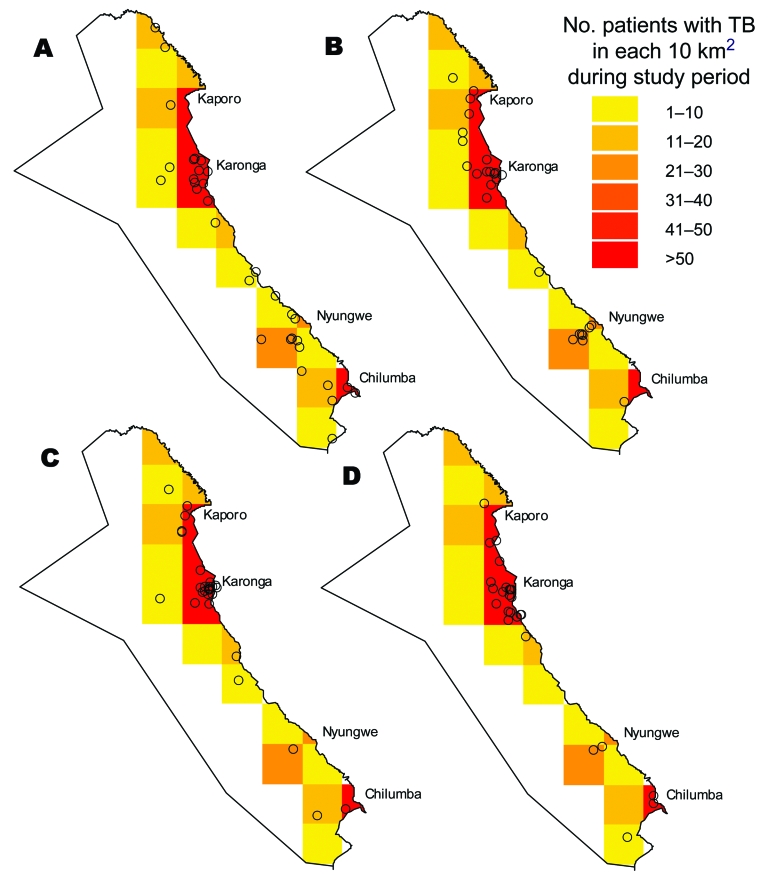
Geographic distribution of the 4 most common strains defined by restriction fragment length polymorphism: A) strain kps12, B) strain kps121, C) strain kps41, and D) strain kps44. Each o represents a patient. Each square is 10 km × 10 km. The background shading represents the total number of tuberculosis (TB) cases in each area during the study period, which largely reflects the population density.

**Table 3:** Proportion of all tuberculosis cases caused by each of the RFLP-defined large cluster strains over time, Malawi*

Strain	No. patients	Tuberculosis cases caused by each strain, % (no.)				p value
		1995–1997	1998–1999	2000–2001	2002–2003	
Kps10	18	2.3 (6)	0.9 (3)	1.7 (5)	2.8 (4)	0.4
Kps12	34	1.5 (4)	3.9 (13)	4.9 (14)	2.1 (3)	0.1
Kps20	15	1.9 (5)	0.9 (3)	1.7 (5)	1.4 (2)	0.7
Kps21	15	0.8 (2)	2.1 (7)	2.1 (6)	0.0 (0)	0.2
Kps41	37	4.2 (11)	3.0 (10)	4.5 (13)	2.1 (3)	0.5
Kps44	29	3.8 (10)	3.3 (11)	1.4 (4)	2.8 (4)	0.3
Kps64	16	0.4 (1)	1.5 (5)	2.8 (8)	1.4 (2)	0.1
Kps97	15	0.8 (2)	2.4 (8)	1.0 (3)	0.7 (1)	0.3
Kps104	16	1.5 (4)	0.9 (3)	2.1 (6)	2.1 (3)	0.6
Kps121	27	4.2 (11)	2.7 (9)	2.1 (6)	0.7 (1)	0.03 (trend)

*RFLP, restriction fragment length polymorphism.

Spoligotypes from large clusters (>15 people) were compared with the international database (*10*,*11*). The results, displayed according to the octal code, are shown in Table 4 (*13*). Six of the large cluster strains had patterns identical or very similar to spoligotype 59, which is classified as ubiquitous and recurrent (*10*,*11*). These 6 RFLP-defined strains (kps10, 12, 20, 21, 41, and 64) had similar RFLP patterns, with a similarity coefficient of 79% (with 1% position tolerance).

**Table 4:** Spoligotypes for the RFLP-defined large cluster strains with at least 5 bands*

Strain no.	No. bands on RFLP	No. examples spoligotyped	Spoligotype octal description	International classification	Comment
Kps41	11	5	777777606060771	59	Widespread
Kps20	8	3	777777606060771	59	Widespread
Kps21	8	2	777777606060771	59	Widespread
Kps10	10	1	777777606060771	59	Widespread
		1	777777206060771	Not recorded	
Kps12	9	3	577777606060771	Not recorded	
Kps64	9	2	777777606060771	59	Widespread
		1	777437606060731	Not recorded	
Kps121	13	2	700777747413771	129	3 recorded, Zimbabwe, French Guiana
Kps104	14	2	703377400001771	21	Widespread
		1	703377400001631	Not recorded	
Kps44	16	5	777777777760771	53	Widespread
Kps97	22	2	000000000003771	1	Widespread, Beijing

*RFLP, restriction fragment length polymorphism.

The spoligotypes for RFLP-defined strains kps104, kps44, and kps97 were also identical or similar to previously described widespread spoligotypes, types 21, 53, and 1 (Beijing), respectively. The spoligotype for strain kps121, spoligotype129, was not similar to any widespread types.

The spoligotypes from the RFLP-defined large cluster strains were compared with spoligotypes from patients with positive cultures in 2002, and from patients with smear-positive tuberculosis and unique RFLP patterns in 1998 through 1999. Overall, 9 (90%) of 10 of the large cluster strains had spoligotypes that were identical to, or only 1 spacer different from, previously described widespread spoligotypes. For the patients from 2002, this proportion was 90 (71%) of 126 (p = 0.3 when compared to the large cluster strains), and for the smear-positive unique strains, it was 37 (66%) of 56 (p = 0.3 compared to the large cluster strains).

All the spoligotypes that were found in the RFLP-defined large cluster strains were also found among (RFLP-defined) unique strains. Seventeen of the unique strains had spoligotype 59, and 2 others had closely related patterns (i.e., 1 spacer different); 1 had spoligotype 21, and 1 had a closely related pattern; 4 had spoligotype 53, and 2 had closely related patterns; and 6 had spoligotype 129. Of the 56 patients from 1998 to 1999, none had Beijing spoligotypes, but we have previously described strains with Beijing spoligotypes and unique RFLP patterns in this population (*14*).

The spoligotypes found in the large cluster strains were also common among the unselected patients from 2002. Thirty-six (29%) had spoligotype 59, and 10 more had closely related patterns; 11 (9%) had spoligotype 21; 8 (6%) had spoligotype 53, and 2 had closely related patterns; 7 (6%) had the Beijing spoligotype; and 8 (6%) had spoligotype 129. The 36 isolates with spoligotype 59 had 23 different RFLP patterns with a similarity coefficient of 63%.

## Discussion

This study suggests that both epidemiologic and strain-related factors may contribute to large cluster size. In large clusters young adults, male patients, and those living in the town were over-represented, all factors likely to be associated with increased social mixing. Similar associations with age and sex have been found previously, in the United States and Denmark. In Denmark the largest cluster was particularly predominant in the capital city (*1*,*2*).

There was no significant association between tuberculosis type (smear positive, smear-negative pulmonary, or extrapulmonary) and cluster size, but most patients had sputum smear–positive disease. There was also no statistically significant association with degree of smear positivity (not shown). An overall association with infectiousness would not necessarily be expected: the infectiousness of the first cases of a cluster may be important in determining size, but the first cases for the large clusters, which were found throughout the period of study, are not identifiable. There was no significant association with isoniazid resistance, but only 39 (6%) patients had resistant strains. Isoniazid resistance has been associated with reduced clustering and reduced generation of secondary cases (*15*,*16*) so it might have been expected to be less common in the larger clusters. Only 3 clustered patients had rifampin resistance in our study (2 with 1 strain and 1 with another), so the effect of this factor on cluster size could not be investigated.

The factors associated with cluster size were not identical to those associated with clustering overall (*5*). Whereas younger adults were more likely to have clustered strains and to be in large clusters, female patients were more likely to have clustered strains but among clustered case-patients, male patients were more likely to be in large clusters. Known contact with a previous tuberculosis patient is an important risk factor for tuberculosis, especially for women in this population (*17*). It may be that women are particularly likely to become infected at home (and therefore be in small clusters) and that men are more likely to become infected outside the home, sometimes from outside the area (seen as unique strains) and sometimes as part of large clusters.

We found no evidence of an association of cluster size with HIV status, although we had previously found HIV to be associated with clustering among older patients (*5*). The effect of HIV infection on clustering is complex since it depends both on the biologic effects of HIV (increasing the risks for active disease—perhaps to different extents for primary and postprimary disease—and decreasing infectiousness) and on any tendency for HIV and tuberculosis to affect the same subpopulations with shared risk factors.

Strain virulence was assessed by examining the proportion of patients who died: there was no association with cluster size either overall, or separately, in HIV-positive or -negative patients (data not shown). Virulent strains could lead to large clusters if virulence were associated with increased transmission rates or increased rates of disease after infection (*3*). However, virulent strains could have less opportunity to transmit if the severity of symptoms leads to early treatment or death, thus reducing the duration of the infectious period.

Evidence that strain characteristics may have contributed to cluster size comes from the finding that the spoligotypes of most of the common RFLP-defined strains in this study were identical to, or only 1 spacer different from, widespread spoligotypes already described. Unique RFLP-defined strains from smear-positive patients in the early part of the study were used as a comparison group. Smear-positive case-patients were chosen to maximize the likelihood of transmission occurring; early cases were used to allow time for secondary cases to have been identified if they had occurred. These unique strains were less likely than the large cluster strains to have spoligotypes that were closely related to widespread types, but this difference was not statistically significant, and the spoligotypes that were found in the large cluster strains were also found among the unique strains. Interestingly, strain kps121, which was the only large cluster strain with a spoligotype not closely related to a widespread previously described type, was also the 1 large cluster strain that was clearly decreasing in the Karonga population.

The finding of large cluster strains with previously described widespread spoligotypes may suggest that these strains are particularly transmissible or particularly likely to cause disease. Other possibilities are that they are older in evolutionary terms, and thus have had more time to become widespread, or that we are seeing a founder effect in some populations with subsequent spread following human migration patterns. Spoligotype 59 was common in the Malawi population in all groups of patients, clustered and unique, and was associated with a wide diversity of RFLP patterns, which suggests that it may be a longstanding strain in this area. It was also the most common spoligotype found in studies in Zimbabwe and Zambia (*18*,*19*). However, spoligotype 59 was particularly common among the isolates from large clusters, with more closely related RFLP patterns, consistent with some variants having high transmissibility. Spoligotype 59 has been classified as belonging to the Latin-American-Mediterranean lineage (*18*), and as part of the strain family Southern Africa Family 1 (*19*). The large cluster strain kps97 had a Beijing spoligotype and in total, we have previously identified 44 patients with Beijing strains in this dataset, with 12 different RFLP patterns (*14*). Beijing strains have been associated with increased virulence and growth rates in vitro (*20*–*22*). That there are true differences in strain characteristics between other clustered and nonclustered strains is beginning to be established in in vitro studies from other populations (*23*).
